# Surface wave tomography using dense 3D data around the Scrovegni Chapel in Padua, Italy

**DOI:** 10.1038/s41598-022-16061-1

**Published:** 2022-07-12

**Authors:** Ilaria Barone, Giorgio Cassiani, Amine Ourabah, Jacopo Boaga, Mirko Pavoni, Rita Deiana

**Affiliations:** 1 Dipartimento di Geoscienze, Università degli Studi di Padova, via G. Gradenigo 6, Padova, 35131 Italy; 2 Centro Interdipartimentale per i Beni Culturali, Università degli Studi di Padova, piazza Capitaniato 7, Padova, 35139 Italy; 3 Stryde, 1-2 Paris Garden, London, SE1 8ND UK; 4 Dipartimento dei Beni Culturali, Università degli Studi di Padova, piazza Capitaniato 7, Padova, 35139 Italy

**Keywords:** Geophysics, Seismology

## Abstract

A dense single-node 3D seismic survey has been carried out around the Scrovegni Chapel in Padua (Italy), in order to give new insights about the archaeological setting of the area. The survey made use of nearly 1500 vertical nodes deployed over two rectangular grids. 38 shot positions were fired all around the two receiver patches. The fundamental mode Rayleigh wave signal is here analysed: traveltimes are directly inferred from the signal phases, and phase velocity maps are obtained using Eikonal tomography. Also surface wave amplitudes are used, to produce autospectrum gradient maps. The joint analysis of phase velocity and autospectrum gradient allowed the identification of several buried features, among which possible remains of radial walls of the adjacent Roman amphitheater, structures belonging to a medieval convent, and the root area of an eradicated tree. Finally, depth inversion of 1D dispersion curves allowed the reconstruction of a quasi-3D shear-wave velocity model.

## Introduction

The Scrovegni Chapel is worldwide famous for the fresco cycles painted by Giotto, recently inserted in the UNESCO World Heritage List as a part of “Padua’s fourteenth-century fresco cycles”. The Chapel stands on the remains of a Roman amphitheater. In particular, the building has two wholly separated levels, one above and the other below the surface (hypogeum). Below the main facade, the south-western wall of the hypogeum corresponds to one of the elliptical walls of the Roman amphitheater. During the centuries, the construction of different buildings and their buried remains affected this complex archaeological area, as evidenced by historical documents^1^.

Today, we only have poor elements to thoroughly understand the exact role of the hypogeum in the Chapel and its relation with the buried undiscovered remains of the Roman amphitheater, among other most recent remains distributed in this area^2^. From an archaeological point of view, several excavations carried out between 1880 and 2013 brought to light different elements, such as a system of corridors among three concentric elliptical walls, a possible gallery running along the minor axis of the amphitheater, the remains of about 3 m thick concrete slab foundation and a few standing radial walls on the south-western side. No evidence of expected galleries along the central axis of the amphitheater has ever been found, although their existence has been hypothesized, and no new remains of radial walls in other locations confirm their supposed orientation, apparently tilted with respect to the longitudinal walls of the Chapel^1^.

This study aims at giving new insights into the knowledge of this area, with a specific focus on the buried remains of the Roman amphitheater. This objective is here achieved through the analysis of seismic surface waves, in particular by applying surface wave traveltime tomography to a dense 3D seismic dataset. Surface wave tomography (SWT) is a widespread technique, originally used in global seismology to image crustal and upper mantle structures. At that large scale, the active sources used for the inversion are natural seismic events^3–6^ or low-frequency seismic ambient noise^7–10^. SWT applied to small-scale near-surface data is recently gaining popularity, because of its capability to resolve shallow lateral velocity variations. In most cases, small scale applications make use of active (controlled-source) data^11–17^ or passive records^18–20^ originally acquired for exploration purposes, since they guarantee a dense receiver coverage. Small scale applications specifically designed for SWT are very rare^21–24^. More specifically, at the time of this writing, no SWT study has ever been carried out for archaeological prospection, where the required resolution is metric to sub-metric.

In this study we perform a 3D dense single-node seismic survey, including both active and passive measurements, with the main purpose of analysing the Rayleigh wave propagation. In particular, this piece of work focuses on the analysis of the active seismic data. Both surface-wave phases and amplitudes are processed in parallel, since their joint analysis is capable of imaging both smooth and sharp lateral velocity variations. Depth inversion of local dispersion curves is finally applied to derive a high-resolution quasi-3D shear-wave velocity model of the near surface, to be used for archaeological interpretation.

## Acquisition scheme

**Figure 1:**
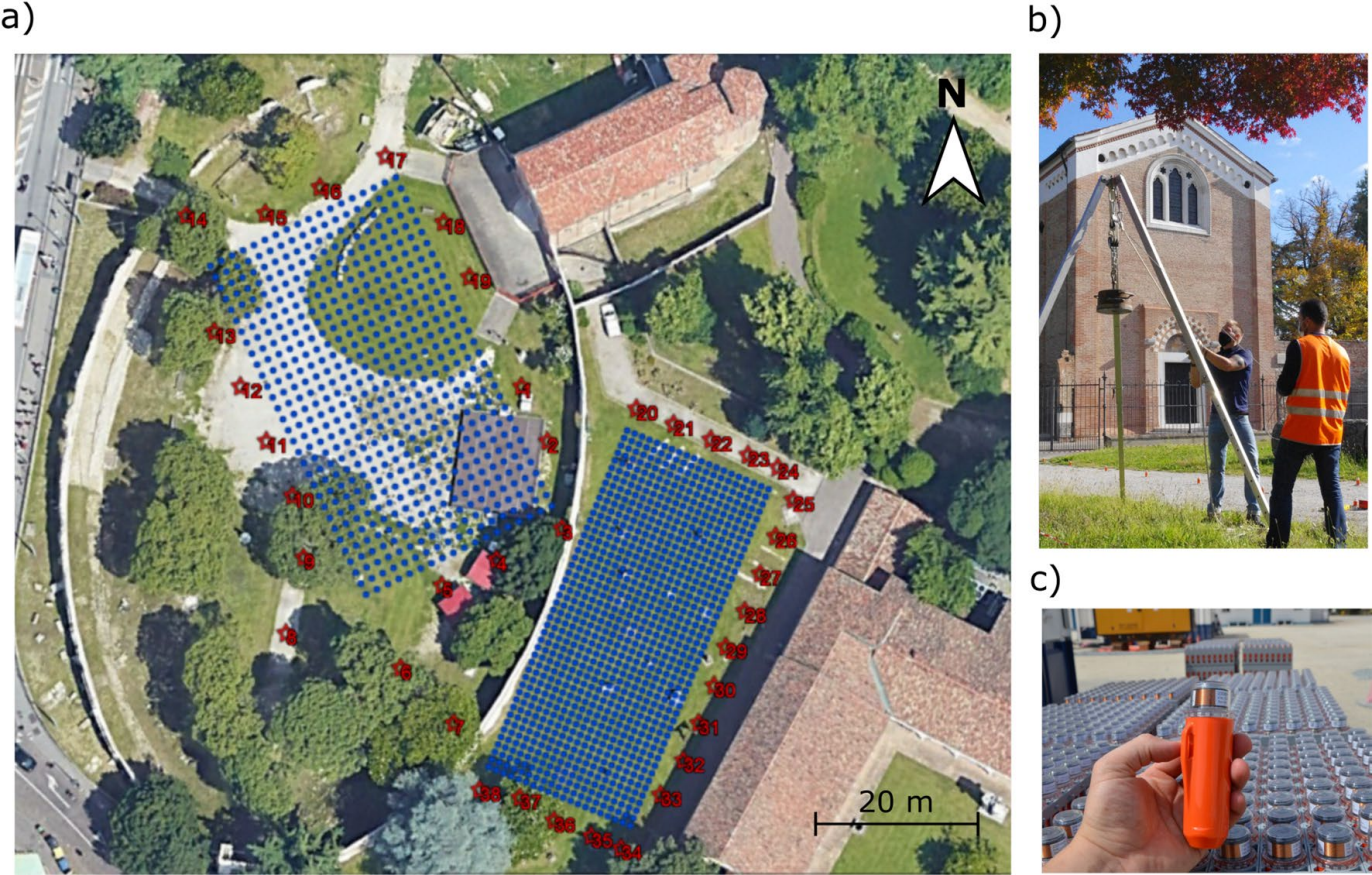
(**a**) Acquisition scheme for the 3D active/passive campaign. The blue points represent the locations of the autonomous seismic nodes, the red stars the locations of the active seismic sources. Note the well-preserved elliptical wall of the amphitheater and the Scrovegni Chapel on the Northern-Eastern side. (**b**) Weight drop source operating in front of the Scrovegni Chapel. (**c**) Autonomous seismic nodes used for the acquisition.

The seismic acquisition includes both active and passive recordings. 1473 autonomous seismic nodes^25^ (Fig. 1c) were placed vertically over two rectangular grids of equal spacing in both spatial dimensions (Fig. 1a). The grids have different orientations to optimize the coverage of possible relevant features, e.g., the presumed gallery along the major axis of the amphitheater and the radial walls on the southern-eastern side, and of known archaeological elements, such as the system of galleries along the minor axis and remains of a medieval convent (the Eremitani convent). Also the spacing between sensors has been adapted to the degree of resolution required to image the structural elements of interest, being 1.5 m for the 25.5 m $\times$ 45 m receiver patch inside the amphitheater (hereinafter we will refer to it as “grid 1”), and 1 m for the patch outside, of size 19 m $\times$ 45 m (“grid 2”). Due to the presence of obstacles in the area, five nodes are missing from their theoretical positions, others have been moved a few centimeters apart (we estimated positioning errors within a ± 10 cm range).

The nodal system can measure vertical accelerations in a frequency band of 1–125 Hz with a flat response in both phase and amplitude. The small size and weight of the sensors (13 cm $\times$ 4 cm, 150 g) and the absence of cables allowed very rapid field operations. The nodes recorded for about 22 h continuously, thus collecting both active and passive signals. As for the active acquisition, 38 shot locations around the two receiver patches were designed to produce a uniform azimuth distribution (Fig. 1a). The source was a weight drop, with a metallic disc of a 70 kg falling from a height of 1.5 m (Fig. 1b). Each location was energized two to four times, stacking to increase the signal-to-noise ratio.

In addition, a tri-component velocimeter and accelerometer was placed at the base of the main facade of the chapel in order to monitor the accelerations induced by the active source, which never exceeded $10^{-4} g$ (the gravitational acceleration).

## Results

**Figure 2:**
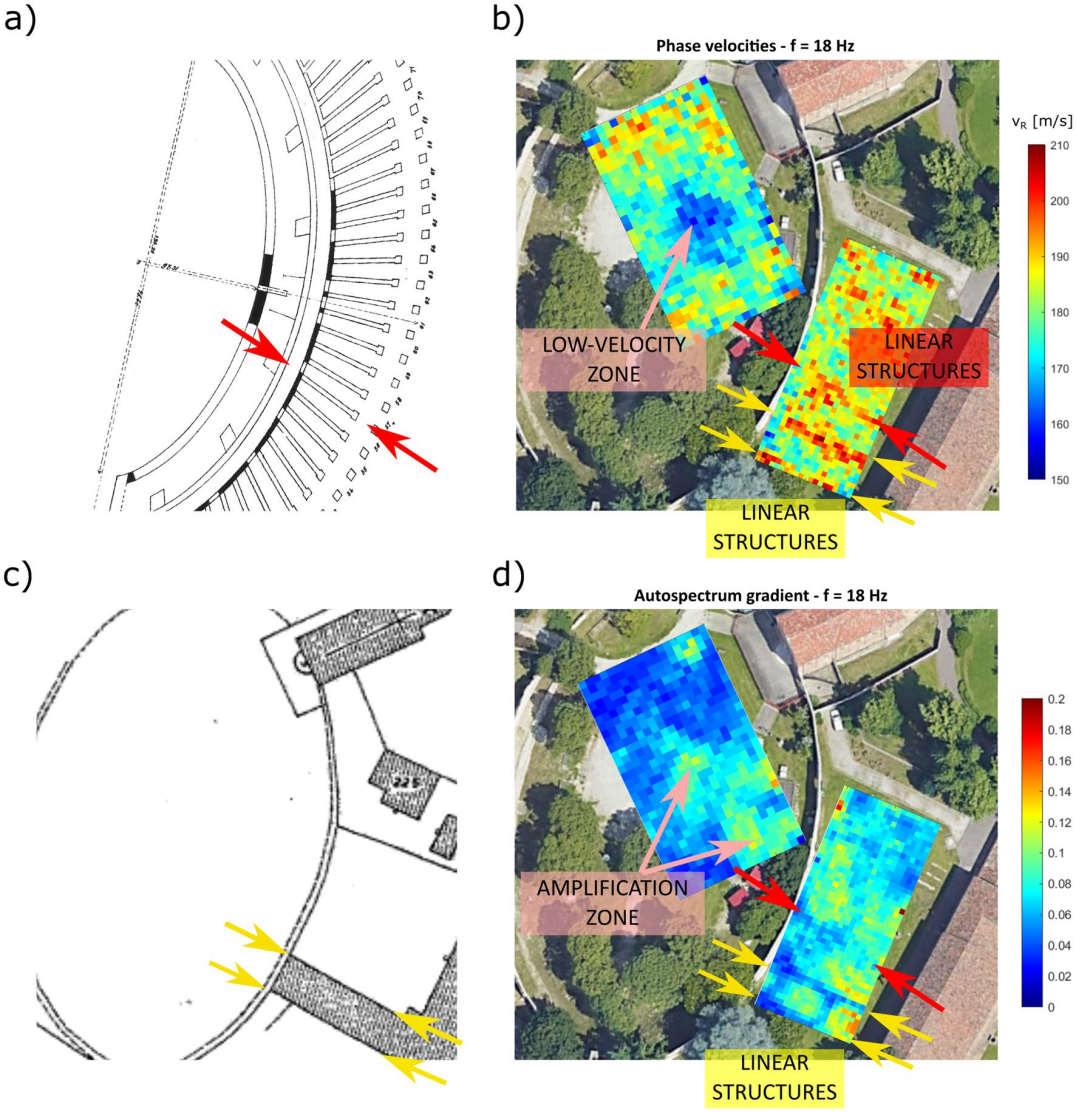
(**a**) Detail of Brunelli Bonetti’s hypothetical reconstruction of the amphitheater (modified from Bressan^27^). (**b**) Phase velocity map for 18 Hz. (**c**) Cadastral map of Padua dating back 1966. (**d**) Autospectrum gradient map at 18 Hz. The corresponding features are highlighted with arrows of the same color.

**Figure 3:**
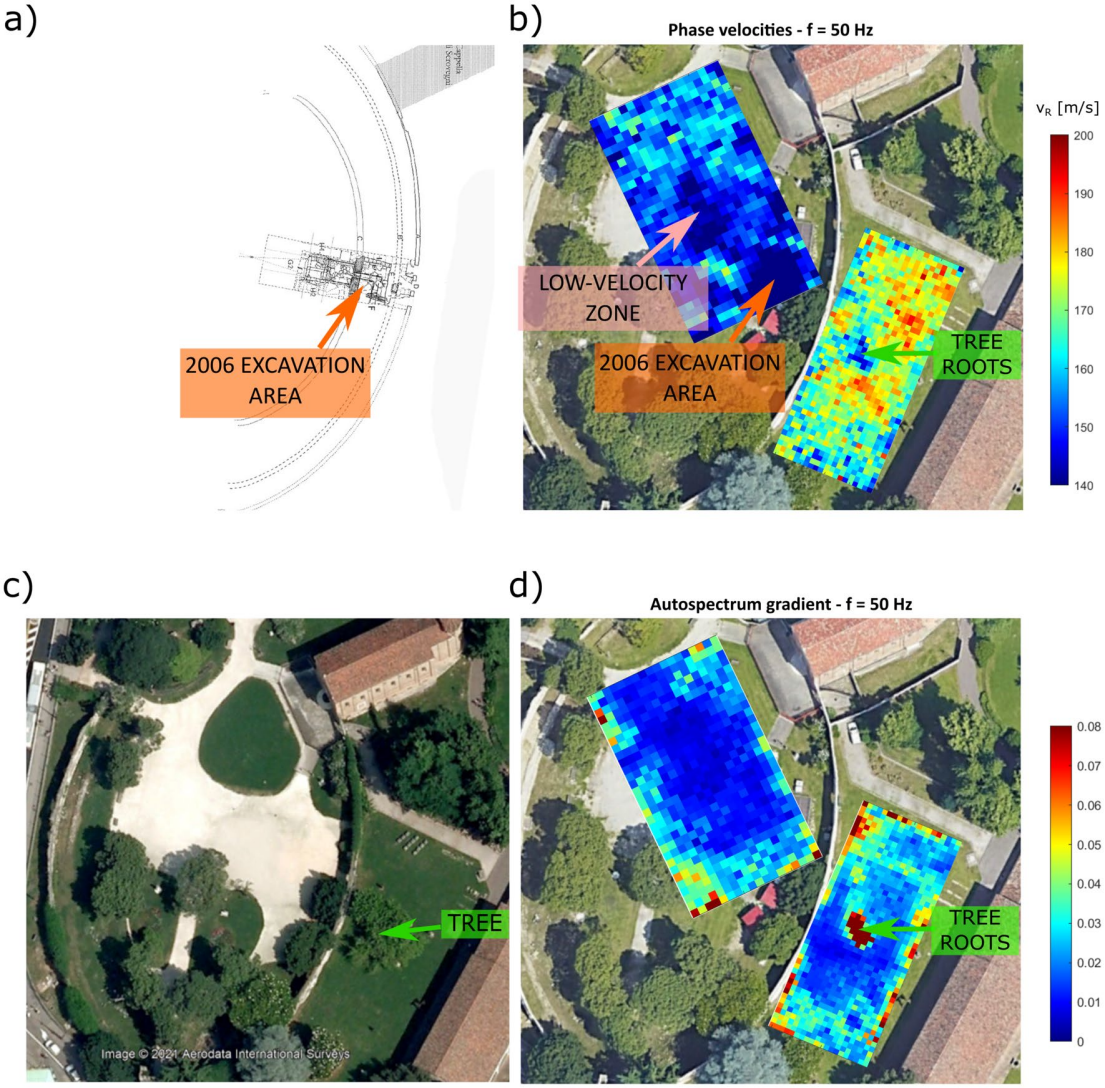
(**a**) Detail of the reconstruction of the amphitheater given by Ruta et al.^28^, including the 2006 excavation area (modified from Bressan^27^) (**b**) Phase velocity map for 50 Hz. (**c**) Satellite image dating 2009 (from Google Earth). (**d**) Autospectrum gradient map for 50 Hz. The corresponding features are highlighted with arrows of the same color.

The analysis of surface-wave phases and amplitudes described in the section “Methods” brings to the retrieval of the phase velocity and autospectrum gradient distributions for all frequencies of analysis. Figures 2 and 3 show the phase velocity and autospectrum gradient maps for 18 Hz and 50 Hz, for grid 1 and grid 2. Note how the overall velocities are higher outside the amphitheater wall, suggesting different subsoil conditions. The average relative error is about 10%, and rarely exceeds 20% (see supplementary information, Text S6). This is a reasonable value given the low number of shots averaged. In this regard, Lin et al.^19^ show how the standard deviation significantly decreases with the number of shots included in the analysis. Velocity values distributed at the borders of each area are characterized by higher uncertainties, due to a lower data coverage. In addition, the error associated to mis-positioning of the receivers (up to 6.7% for grid 1 and 10% for grid 2, considering a maximum error of 10 cm) brings to systematic errors that add up to the estimated uncertainties.

The maps at different frequencies highlight different features, because of their different sensitivity with depth. At 18 Hz we recognize some patterns both in the phase velocities and energy gradient maps: some high velocity alignments outside the amphitheater wall appear, and further structures perpendicular to them clearly show up. Part of these alignments can be traced back to a side structure of the Eremitani convent, attached to the amphitheater wall, and visible in Padua city cadastral maps until 1966 (Fig. 2c), while others seem aligned with the supposed orientation of the radial walls given by Brunelli Bonetti^26^ (Fig. 2a). In the middle of grid 1, a low velocity zone strongly correlate with higher values of the autospectrum gradient. In this case, the velocity anomaly could be explained with the former presence of an artificial pond, displayed in some historical pictures. At 50 Hz, the phase velocity distribution in grid 1 shows again lower velocities in the middle of the area, but also a low-velocity area that corresponds to the archaeological excavation of 2006 (Fig. 3a). These features are not clearly recognizable in the autospectrum gradient maps. As for grid 2, a delimited low-velocity area strongly correlates with a marked anomaly in the autospectrum gradient, that coincides with the root area of a former tree, removed between 2011 and 2013 (Fig. 3c): the tree eradication likely caused a decrease of soil density, with a consequent reduction of phase velocities and the occurrence of amplification phenomena. For the sake of completeness, phase velocity maps and energy gradient maps for all frequencies of analysis are displayed in the supplementary information (Supplementary Text S5).

**Figure 4:**
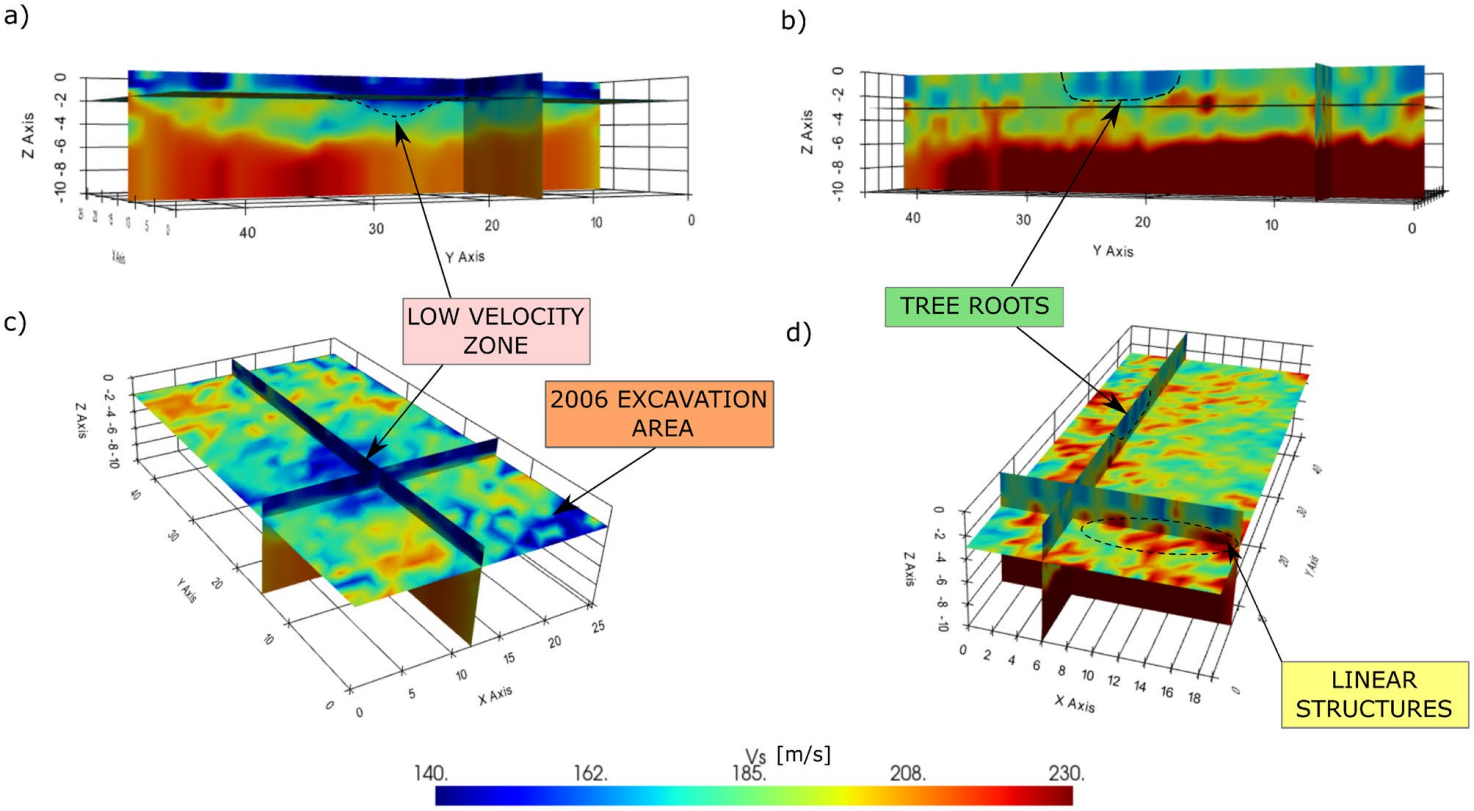
Quasi-3D $V_s$ model obtained through depth inversion of local dispersion curves in grid 1 (left) and grid 2 (right). (**a**,**b**) View from East. (**c**,**d**) View from South with 30 degrees elevation.

The final quasi-3D models for grid 1 and grid 2, obtained after depth inversion of local dispersion curves, are shown in Fig. 4, allowing to estimate the true depths of the different structures. The tree root zone only affects the first two meters, as expected. The linear structures identified in the area outside the amphitheater are located at an approximate depth of 3 m, which is consistent with the expected depth. The low-velocity area inside the amphitheater extends down to 3 m depth, and its shape is compatible with the presence of a man-made pond. As for the deeper part of the model, in both areas a sudden increase of $V_s$ is observed around 6 m, which likely indicates a change in the soil stratigraphy, reaching the bottom of the anthropic layer. However, velocities deeper that 6 m are higher outside the amphitheater, indicating again different subsoil conditions for the two areas.

## Discussion

A striking correspondence between phase velocity maps and autospectrum gradient maps is observed at most frequencies: in many cases clear high-velocity and low-velocity anomalies correspond to amplitude variations. However, some of the observed features are only visible in one of the two maps. This is because of the complementarity of the information brought by phase (smooth velocity variations) and amplitude (sharp lateral variations, boundaries between different materials, etc.). For this reason, the joint analysis of phase velocity and autospectrum gradient maps is a critical step for a correct interpretation of the results. The quasi-3D $V_s$ model obtained after depth inversion of phase velocities is therefore a smooth representation of the true velocity distribution, which lacks of all small scale features, especially at large depths. However, the inverted model allows a correct interpretation of the depths at which the previously identified structures are.

The present study is successful in imaging small-scale structures at the archaeological scale with the analysis of surface waves only. This has been possible thanks to a very dense 3D single-node acquisition, which allowed a regular and fine sampling of the wave propagation, overcoming the limitations due to the high level of noise in the data. In fact, the a posteriori tests on resolution (see Supplementary Text S7) demonstrate the possibility of imaging very small structures (2–3 times the receiver spacing) when the velocity contrast with the surrounding medium is at least 20%. However, with lower velocity contrasts, only bigger scale anomalies are detectable. For this reason, the current model can truthfully represent only strong anomalies, while smaller scale structures or smoother variations are ignored.

Known archaeological features were clearly imaged, such as the system of galleries discovered by the archaeological excavation of 2006, appearing as a low-velocity anomaly, and remains of the Eremitani convent, imaged both as high-velocity linear anomalies and autospectrum gradient anomalies. In particular, the high resolution and continuity of the Eremitani convent structures tell us about the good state of preservation of these remains, an element about which we did not have previous information.

Some of the initial questions about the structures of the Roman amphitheater have now an answer. We found no evidence of a gallery along the major axis of the amphitheater: this outcome does not entirely exclude its existence, since the gallery could have (partially) collapsed and/or it could have been filled with dense material, indiscernible from the outside material. Linear structures were found outside the amphitheater on the southern-eastern side, oriented as the supposed direction of radial walls. It is plausible that these structures are remains of partially-preserved radial walls, or remains of later structures of which we do not have any record.

Finally, some unexpected features, only partially connected to the archaeological character of this survey, were found. The low-velocity zone imaged inside the amphitheater could be hardly attributed to a structure of the amphitheater, both because of its size and position. Instead, the presence of a former pond in that area is confirmed by historical pictures dating back to the early twentieth century, when the interior of the amphitheater was a garden. The sharply defined low-velocity anomaly outside the amphitheater wall, undoubtedly attributed to the root zone of an eradicated tree, is extremely clear both in terms of shape and phase-velocity/autospectrum gradient amplitudes, in spite of its limited size. This confirms the strong potential for surface wave analysis to detect small-size shallow velocity anomalies, suggesting the opportunity to use this methodology in different fields, such as civil engineering, soil science and biology.

## Methods

The processing scheme of the raw 3D data is similar to the one proposed by Barone et al.^17^. Traveltimes for different frequencies are directly extracted from the phase of the surface wave signal, taking advantage of the fine sampling in both spatial directions. However, the phase distribution needs to be representative of only one mode of propagation (i.e., the Rayleigh wave fundamental mode). More generally, any coherent noise (e.g. higher modes, vibrations from fixed noise sources, backscattering, etc.) needs to be removed from the signal, since it perturbs both phase and amplitude variations with offset, generating periodic patterns, as explained in detail by Barone et al.^29^.

Preliminary analyses on the recorded dataset are needed to study the characteristics of the signal, including the frequency content of Rayleigh waves, the presence of higher order modes and/or of coherent noise and the degree of heterogeneity in the two survey areas, and to identify the LMO velocities used for the correction in the 3D processing (see Supplementary Text S1). This preliminary work consists in the analysis of f–k spectra computed over several 2D lines extracted from the 3D dataset. Results highlighted substantial backscattered energy and at least one higher order mode of propagation with non-negligible energy. The frequency band interested by the fundamental mode extends from 10 to 50 Hz, making this interval suitable for our later analysis. Dispersion curves picked along the different lines show a high degree of dispersion, revealing very heterogeneous conditions. From this analysis we can also appreciate the overall lower velocities inside the amphitheater with respect to the area outside, which has been clearly illustrated in the “Results” section.

The 3D processing sequence described below refers to a single shot and a single frequency and consists of three main steps. First, we apply a linear moveout (LMO) correction to raw traces to (i) reduce phase jumps related to the 2$\pi$ periodicity of the phase, and (ii) move higher modes energy to the negative wavenumber quadrant of the f–k spectrum. Second, we perform pseudo-2D f–k filtering over azimuthal sectors of equal width. Third, we apply a 2D phase unwrapping scheme to compensate for residual phase jumps and we remove the LMO. Frequencies used in this analysis are 10.00 Hz, 10.98 Hz, 12.16 Hz, 13.64 Hz, 15.52 Hz, 18.00 Hz, 21.43 Hz, 26.47 Hz, 34.62 Hz and 50 Hz. Such an inversely-regular frequency sampling reflects into a quasi-regular sampling in depth. In fact, the depth of maximum sensitivity of surface waves is proportional to the wavelength, which is inversely proportional to frequency. The obtained phase maps are finally converted into relative traveltime maps (referred to the receiver closest to the source) as:

1 $$\begin{aligned} \Delta t = \frac{\phi - \phi _0}{2 \pi f}, \end{aligned}$$

where $\Delta t$ is the relative traveltime, $\phi$ is the phase recorded at any receiver, $\phi _0$ is the phase recorded at the receiver closest to the source and *f* is frequency. Two separate processing stages were run for grid 1 and grid 2, using local coordinates. Only close shots around each patch, covering all azimuths, were selected: shots 1–19 were included in the processing of grid 1 while shots 2–7 and 20–38 were used for grid 2. A detailed description of the different processing steps is given in the supplementary information (Supplementary Texts S2–S4).

The traveltime maps for one frequency and all shots are input into an Eikonal Tomography scheme to extract phase velocity maps. Eikonal tomography derives phase slownesses at one frequency from the traveltime gradient magnitude^9^. This operation is repeated for each shot point, and phase velocities are finally computed as the inverse of the average phase slownesses over the different shots. This tomography method is very fast since it does not involve a true inversion (see discussion in Barone et al.^17^). Moreover, the regular receiver geometry of our dataset is perfectly suitable for this method, since it makes unnecessary the most critical and user-dependent step of Eikonal Tomography, which is the 2D traveltime interpolation over a regular grid. Another relevant aspect of Eikonal tomography is that it does not require absolute traveltimes (referred to a source/virtual source position), but it only takes into account traveltime differences between adjacent receivers (for this reason we used relative traveltime maps). Finally, this method permits the extraction of velocity standard deviations, which represent an estimate of the error for the obtained velocities. On the other side, Eikonal tomography is very sensitive to outliers^17^. For this reason, we performed a statistical analysis to identify and remove outliers from single-shot phase velocity maps: velocity values outside the range from spatial mean plus/minus three times the standard deviation have been discarded.

The analysis described so far was mainly focused on the signal phase, with the purpose of extracting velocity information. However, most tomography approaches, including Eikonal tomography, are based on the high frequency approximation^30^. For this reason, the obtainable lateral resolution is intrinsically limited by the signal wavelength which, for the lower frequencies, is much higher than the sensor spacing. The analysis of signal amplitudes may help detect sudden lateral velocity variations caused by small size objects or discontinuities^31^. Several methods to measure amplitude variations are available. We focused on the autospectrum method^32^, which analyses the autospectral density at each frequency *G*(*f*), defined as:

2 $$\begin{aligned} G(f) = \{ Im[Y(f)] \}^2 + \{ Re[Y(f)] \}^2 = \{ A(f) \}^2, \end{aligned}$$

where *Y* and *A* are the complex spectrum and amplitude spectrum of a seismic record, respectively. The autospectral density is a measure of the surface wave energy for a certain frequency: significant spatial variations of this parameter could indicate the presence of a scatterer (negative variation) or an amplifying zone (positive variation)^31^. For this reason, this study focuses on the analysis of autospectrum gradient maps. The procedure followed here consists in the retrieval of autospectral density maps for all shots, which are normalized by their maximum value. Then, the autospectrum gradient is computed, and an average is computed from the gradient magnitude maps from different shots. From this analysis we obtain a robust estimate of the energy spatial variations, even though the positive/negative sign of the anomalies is not preserved. Traces included in the analysis should not be filtered, because they should include backscattering. Moreover, geometrical spreading effects should be removed before Fourier transformation, and the near-offset region should be excluded as for the phase velocity analysis.

Depth inversion is necessary to produce a quasi-3D shear-wave velocity model of the near-surface. In order to ensure inversion stability, phase velocity maps were initially smoothed with a Gaussian filter. The length of the filter is frequency-dependent: we used $\lambda /2$ as filter length, with $\lambda$ being the average wavelength at a specific frequency. By doing so, lower-frequency maps, characterized by a lower spatial resolution, undergo stronger filtering, while higher-frequency maps better preserve their spatial variability. Local dispersion curves were obtained from the superposition of the smoothed phase velocity maps at different frequencies. Dispersion curves at each location were independently inverted, with no lateral constrain. For inversion, we used the dinverdc module inside the Dinver framework of GEOPSY^33^, that uses the neighborhood algorithm^34^. All details about the model space parameterization are given in the supplementary information (Supplementary Text S8).

## Supplementary Information

Supplementary Information.

## Data Availability

The raw seismic dataset used for this research are available in the Research Data Unipd repository (DOI: 10.25430/researchdata.cab.unipd.it.00000557).
