# Supplementary material for: Surface wave tomography using dense 3D data around the Scrovegni Chapel in Padua, Italy
Source: Sci Rep. 2022 Jul 12;12:11806. doi: 10.1038/s41598-022-16061-1 (PMC9276776; doi:10.1038/s41598-022-16061-1)
Supplement: Supplementary file 1 — Supplementary Information. [file 41598_2022_16061_MOESM1_ESM.pdf]

# Supplementary information for "Surface wave tomography using dense 3D data around the Scrovegni Chapel in Padua, Italy"

**Contents of this file**

1. Text S1 to S8
2. Figures S1 to S14
3. Table S1

## Introduction

This file contains a detailed description of the different steps of data processing, in order to extract Rayleigh-wave traveltimes. The full processing has been performed using original Matlab codes written by Ilaria Barone. Supplementary information also includes details about model uncertainties, and a posteriori tests on the obtainable resolution. Finally, it describes the parameters used for depth inversion of dispersion curves. This operation, as already stated in the main body of the article, is performed with Dinver, an open-source tool within the GEOPSY package[1]. The description of both data processing and dispersion-curve inversion is accompanied by figures illustrating the intermediate-step results.

## Text S1. Preliminary analysis

In order to perform preliminary analyses on the raw dataset, it was necessary to define a unique numbering for receiver lines inside the 3D scheme. The receiver line direction was taken as parallel to the longest side of each rectangle, which is South-East/Nort-West for receiver grid 1 and South-West/Nort-East for receiver grid 2. Numbering increases from West to East: we obtain 18 receiver lines in grid 1 (lines 1 to 18) and 20 receiver lines in grid 2 (lines 19 to 38). The analysis discussed in this section refers to all receiver lines aligned with two shot points, one at each line end, which homogeneously cover the two areas of investigation. The analyses for grid 1 and 2 were performed independently.

Seismograms and f-k spectra referring to symmetrical shots inside and outside the arena are shown in Figures S1a-d and Figures S2a-d, respectively. A manual picking of each spectrum was performed to retrieve the dispersion relation for both the fundamental mode and higher modes (when clearly identifiable) for all eight shots of grid 1 and ten shots of grid 2 (Figure S1e and Figure S2e). We finally picked a dispersion curve which clearly separates fundamental modes from higher order modes for all curves (thick black line in Figure S1e and Figure S2e). This curve will serve as an LMO velocity versus frequency relation in the 3D processing (see below).

## Text S2. Linear moveout correction

The linear moveout correction consists in a time shift of the recorded traces. The amount of shift has a linear dependence on the source-receiver distance (offset), being $\Delta t = x/V_{LMO}$, where $x$ is the offset and $V_{LMO}$ is the velocity used for the correction. This simple operation flattens linear events with a velocity of $V_{LMO}$, while events characterized by higher velocities acquire a negative moveout. LMO is here applied to produce a separation in the spectral domain between the fundamental mode and coherent noise (including higher modes). In particular we use the two dispersion relations, one for each receiver patch, derived in the “Preliminary analysis” section (thick black curves in Figure S1e and Figure S2e) as LMO velocity curves as a function of frequency: after the correction, fundamental mode energy at the frequency of analysis projects in the first frequency-wavenumber quadrant and higher modes in the second quadrant of the f-k plot (Figure S3). It is important to stress how the LMO correction does not alter the position of the backscattered energy in the spectral domain, which remains in the second quadrant.

## Text S3. Pseudo-2D f-k filter

Considering to the 3D nature of the acquisition and the regularity of the receiver geometry, we applied a pseudo-2D f-k filter on azimuthal sectors of 5 degrees from the source position (Figure S4). For each slice, the true source-receiver distance is measured. Then, seismic traces are linearly interpolated to regularly sampled offsets and the f-k spectrum is computed. The filter sets spectral amplitudes to zero in the positive-frequency/negative-wavenumber quadrant. Finally, the filtered seismogram is retrieved through a double inverse Fourier transform and linear interpolation to the original locations. Azimuthal sectors with less than 10 traces are discarded from the analysis, as well as the near-offset region, that we consider to correspond to half wavelength. Figures S3e-f show the effect of the filter, both in the space-time and frequency-wavenumber domains: note how both higher modes and backscattering are effectively removed, while the fundamental mode energy is preserved.

The effectiveness of the adopted filter is also confirmed by its ability to remove phase and amplitude oscillations, which are here mainly due to backscattering effects (see the effect of the filter on the signal phases in Figures S5b-c). If Barone et al.[2] described this phenomenon as the result of the interference between two different modes of propagation, the periodic patterns observed here in the raw signal phases and amplitudes must be attributed to the interference between the incoming surface wave and the reflected wave from the arena middle wall. Under these conditions the oscillating period $\Delta X$ is:

$$\Delta X = \frac{2\pi}{k_i - k_r} = \frac{\pi}{k_i} \tag{1}$$

where $k_i$ and $k_r = -k_i$ are the wavenumbers of the incoming and reflected waves. After the application of the filter,

no periodic behaviour is observed anymore, i.e. only one coherent signal (the incoming wave) and one mode of propagation are preserved.

**Text S4. 2D phase unwrapping**
The LMO correction already removes most of phase jumps related to the $2\pi$ periodicity of the phase. However, the LMO velocities used for the correction overestimate fundamental mode velocities, and this results in an under-correction. Moreover, the independent application of the filter for different azimuthal sectors could introduce artifacts that translate into phase jumps at the borders between sectors. For the reasons above, residual phase unwrapping is required.
We adopted a 2D unwrapping algorithm consisting of an iterative procedure. At each iteration, a phase-quality factor is computed as the sum of the variances of the phase derivatives in the X and Y directions in a close neighborhood of each point. The point with the best quality (lower variance) is selected, phase jumps larger than $\pi$ are compensated in four directions (X, -X, Y, -Y) and the point is marked as “unwrapped”. The unwrapping stops when all data points are unwrapped.
After unwrapping, phases are de-LMO corrected and the relative traveltime maps are obtained using equation 1 of the main document. Figure S5 summarizes the different processing steps, showing their effect on surface wave phases and traveltimes.

**Text S5. Phase velocity maps and autospectrum gradient maps**
For the sake of completeness, we show here the phase velocity maps and the autospectrum gradient maps obtained for all frequencies of analysis (Figures S6, S7, S8, S9).

**Text S6. Phase velocity uncertainties**
Eikonal tomography estimates phase velocity uncertainties as velocity standard deviations over the different shots. S10 shows the phase velocities for grid 1 and 18 Hz obtained from shot 1, the final phase velocities for the same frequency, obtained as the average of phase velocity maps for shots 1 to 19, the associated uncertainties and the number of measurements used in the analysis. It is important to stress that Figure S10b is smoother compared to Figure S10a, the latter being statistically unreliable. However, the two figures show the same major patterns, with a low velocity area in the centre. The number of measurements used for the analysis has a distribution that clearly reflects the excluded near-offset region and the side regions discarded by the f-k filter (visible also in Figure S10a), with the maximum coverage in the centre. As a consequence, standard deviations are also lower in the centre of the area. Finally, Figure S10e shows the relative error distribution, which is centered around 10% and rarely exceeds 20%, as stated in the main body of this paper ("Results" section).

**Text S7. A posteriori resolution tests**
The synthetic tests described in this chapter want to investigate the resolution obtainable from the application of Eikonal Tomography, given the acquisition layout used in the present study and the true (measured) uncertainties on the retrieved phase slownesses, in order to validate the obtained results.
Six different synthetic models (phase velocity maps) were used: models in Figure S11a, c, e are characterized by a constant velocity of 200 m/s and an overlapping checkerboard pattern with 20% velocity variations and anomalies of size 12 m, 6 m and 3 m, respectively. Models shown in Figure S12a, c, e differ from the previous only for having lower velocity variations (10%). Both the checkerboard models and the synthetic traveltimes were generated with FMST[3], using the same position and number of shots/receivers of the true data. We applied Eikonal Tomography to synthetic data, and perturbed single-shot slownesses with normally distributed random errors whose standard deviation is equal to the average slowness errors computed on real data (0.0008 s/m).
The retrieved phase velocity maps are shown in Figure S11b, d, f and S12b, d, f. Tests show how the detectability of small scale structures is strongly influenced both by the grid spacing and by the lateral velocity contrast characterizing the anomaly, and are in a very good agreement with real data. For example, the anomaly given by the tree eradication observed in the survey area outside the amphitheatre is about 6 m wide and has a velocity contrast higher than 20%, which correspond to the model in Figure S11c. Another example is the low velocity area images at the centre of the survey area inside the amphitheatre, which is about 12 m wide and has a velocity contrast higher than 10%, corresponding to the model in Figure S12a. Despite the high level of noise, the synthetic tests demonstrate a high degree of detectability for both anomalies. These considerations on resolution based on synthetic models do not take into account possible limitations given by the frequency content of our data.

**Text S8. Depth inversion**

We adopted a five-layer model (including a halfspace) with constant P- and S- velocities and density inside each layer. The ranges of variability of layer thicknesses and S-velocities are shown in Table S1. In particular, S-velocity ranges, depending on the average phase velocities and associated standard deviations at the different frequencies (Figure S13), are different for grid 1 and grid 2. As for P-wave velocities, they were allowed to vary between twice the minimum $V_s$ and three times the maximum $V_s$ of each layer. Finally, the density has been kept constant for all simulations (Table S1). For each dispersion curve inversion the algorithm generated 20000 models, and computed the associated average phase velocity misfit over frequencies. The final model is the average of the best (minimum-misfit) 1000 models (Figure S14).

## References


1. Manning, T. *et al.* The case for a nimble node, towards a new land seismic receiver system with unlimited channels. *SEG Tech. Program, Expand. Abstr.* 21–25 (2018).
2. Barone, I., Strobbia, C. & Cassiani, G. Multimode multioffset phase analysis of surface waves, a new approach to extend mopa to higher modes. *Geophys. J. Int.* **221**, no. 3, 1802–1819, doi: 10.1093/gji/ggaa106 (2020).
3. Rawlinson, N. FMST: Fast marching surface tomography package. Research School of Earth Sciences, Australian National University, Canberra ACT 0200 (2005).

**Table S1.** Parameters defining the model space for dispersion curve inversion.

| Layer | dz [m] | $V_s$ PATCH1 [m/s] | $V_s$ PATCH2 [m/s] | $\rho$ [kg/m3] |
|---|---|---|---|---|
| 1 | 0.5-2.5 | 130-220 | 160-220 | 1800 |
| 2 | 0.5-2.5 | 130-220 | 160-240 | 1800 |
| 3 | 0.5-2.5 | 150-220 | 160-260 | 1800 |
| 4 | 0.5-2.5 | 150-220 | 160-280 | 1800 |
| 5 | Inf | 180-500 | 180-500 | 2000 |

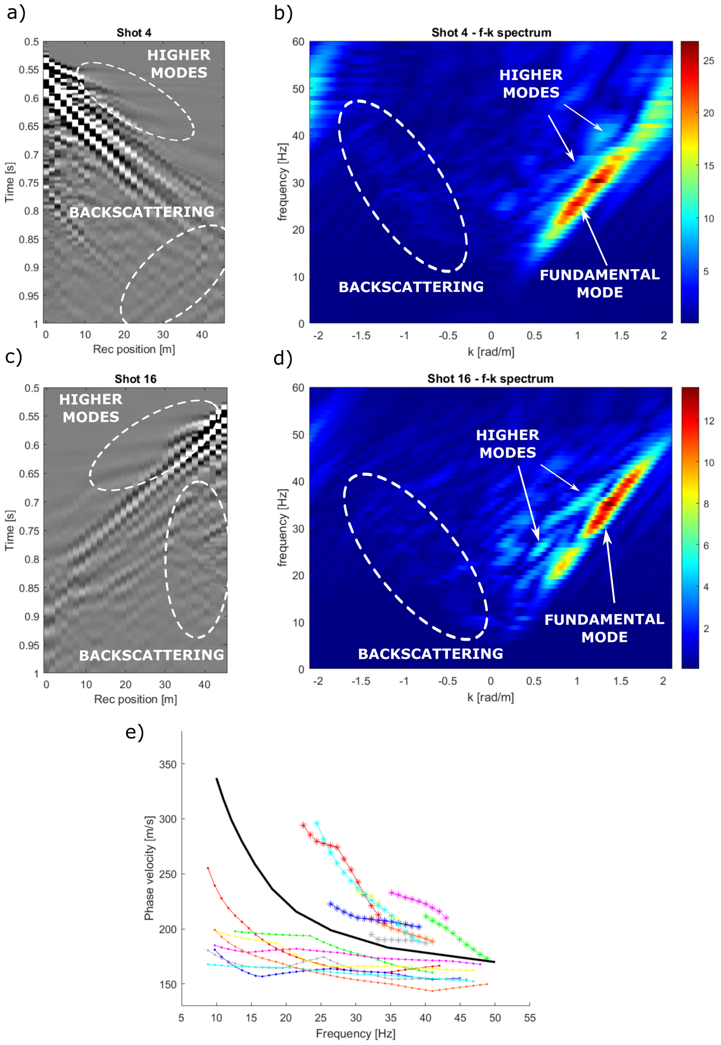


**Figure S1.** (a, c) Examples of reciprocal 2D seismograms extracted from the 3D active dataset acquired inside the arena walls. (b, d) f-k transform of the seismograms in (a) and (c). (e) Picked dispersion curves referring to the fundamental mode (continuous line and point markers) and higher modes (continuous line and asterisks) for the eight shot point/receiver line combinations. The thick black line separating the fundamental mode from higher order modes represents the LMO velocity relation used for the linear moveout correction.

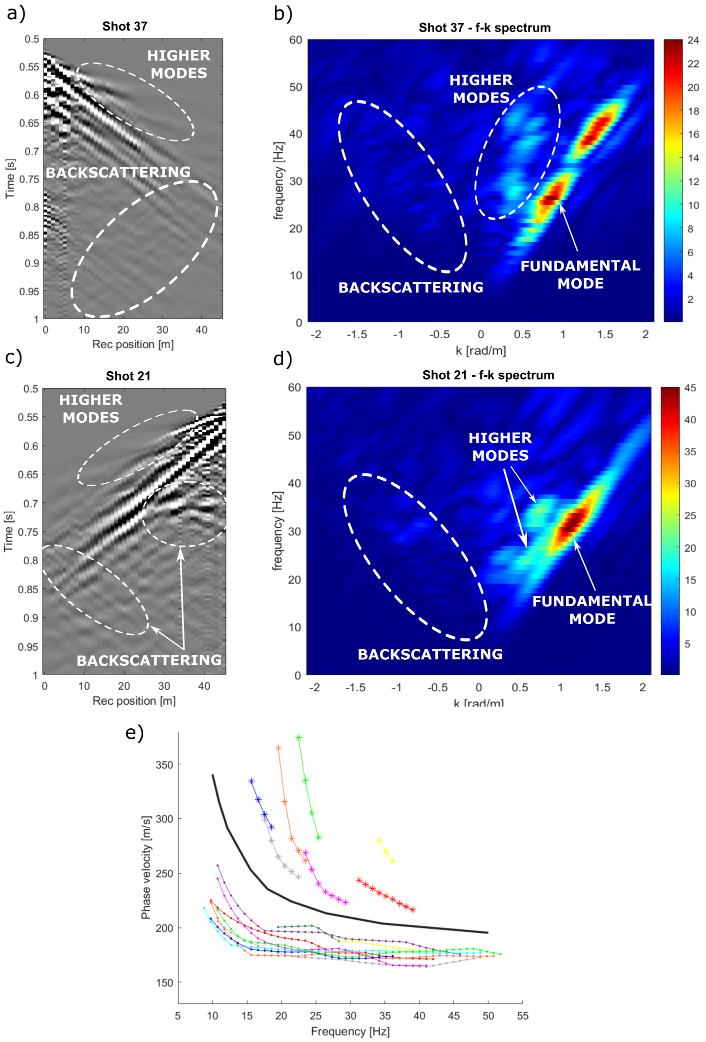


**Figure S2.** (a, c) Examples of reciprocal 2D seismograms extracted from the 3D active dataset acquired outside the arena walls. (b, d) f-k transform of the seismograms in (a) and (c). (e) Picked dispersion curves referring to the fundamental mode (continuous line and point markers) and higher modes (continuous line and asterisks) for the ten shot point/receiver line combinations. The thick black line separating the fundamental mode from higher order modes represents the LMO velocity relation used for the linear moveout correction.

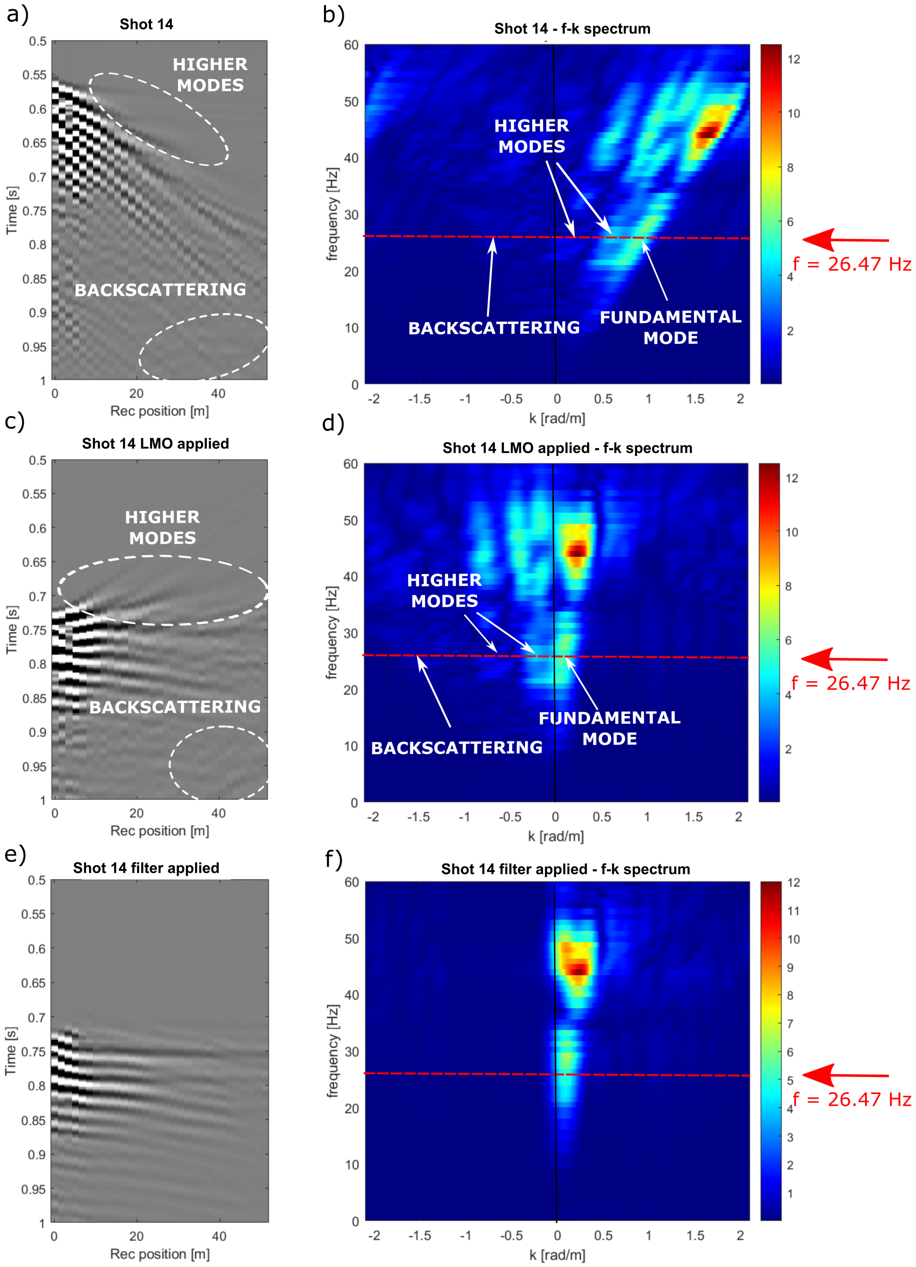


**Figure S3.** 2D seismograms and their f-k spectra referring to shot 14 and receiver line 1: (a, b) raw data, (c, d) data after LMO correction and (e, f) data after pseudo-2D f-k filtering. The analysis refers to a specific frequency ($f = 26.47Hz$, $V_{LMO} = 198.7m/s$). Note how the filter is capable of preserving the fundamental mode energy at that frequency while removing all contributions from higher modes and backscattering.

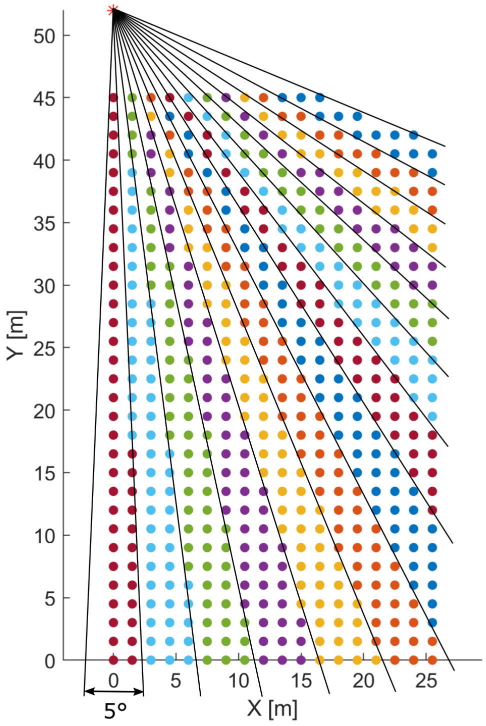


**Figure S4.** Azimuthal sectors for the application of the pseudo-2D f-k filter for shot 14 ($X_s$ = 0 m, $Y_s$ = 52 m) and receiver grid 1. X and Y refer to a local coordinate system with its origin at the southernmost receiver.

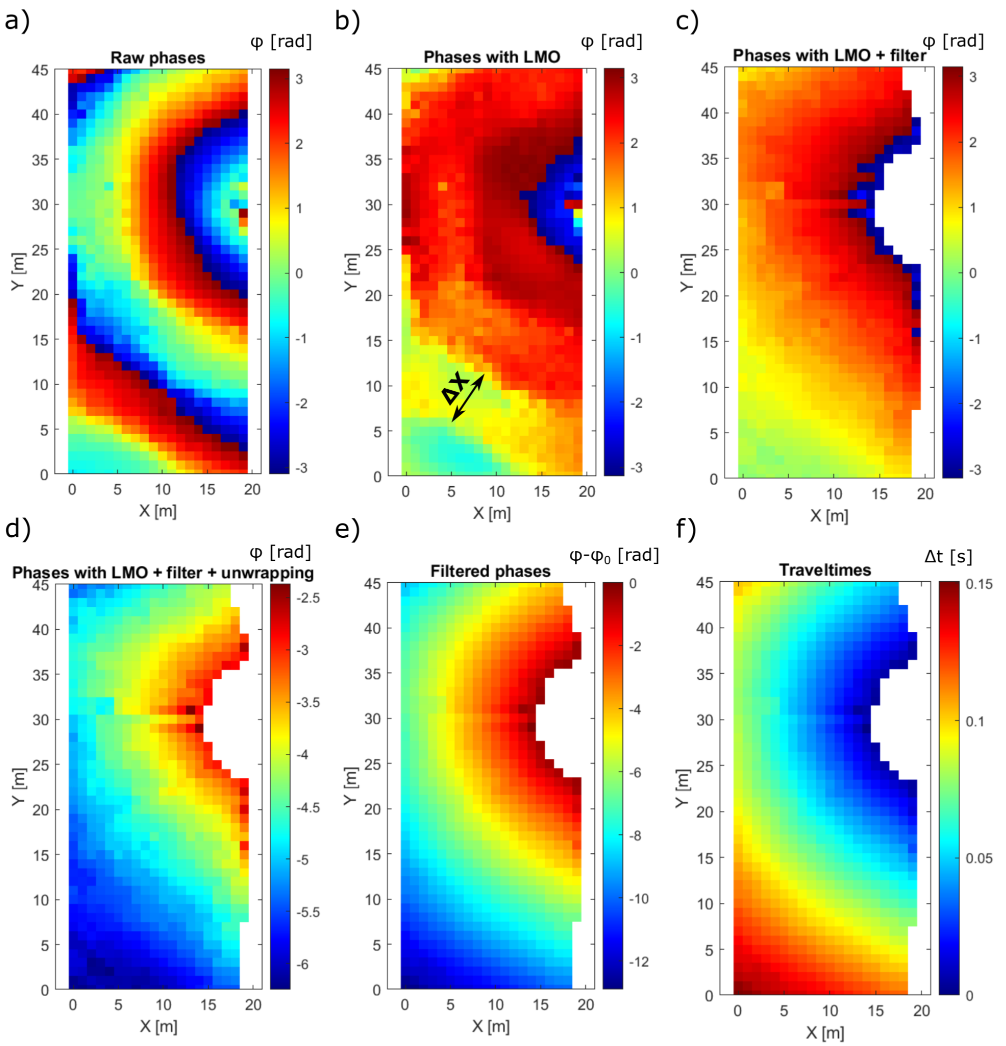


**Figure S5.** Phases/traveltimes for $f = 13.64Hz$, recorded by receiver grid 2 for shot position 28 ($X_s$ = 22 m, $Y_s$ = 30 m). X and Y coordinates refer to a local coordinate system, with its origin at the southernmost receiver. (a) Raw phases. (b) Phases after LMO correction. Note how most $2\pi$ phase jumps are compensated at this stage, while regular phase jumps of smaller entity (with spatial period $\Delta X$) become more evident, which are due to backscattering effects. (c) Phases after the application of the f-k filter. Note the effect of the filter in removing backscattering. (d) Phases after residual 2D unwrapping. (e) Relative phases after de-LMO correction. (f) Relative traveltimes.

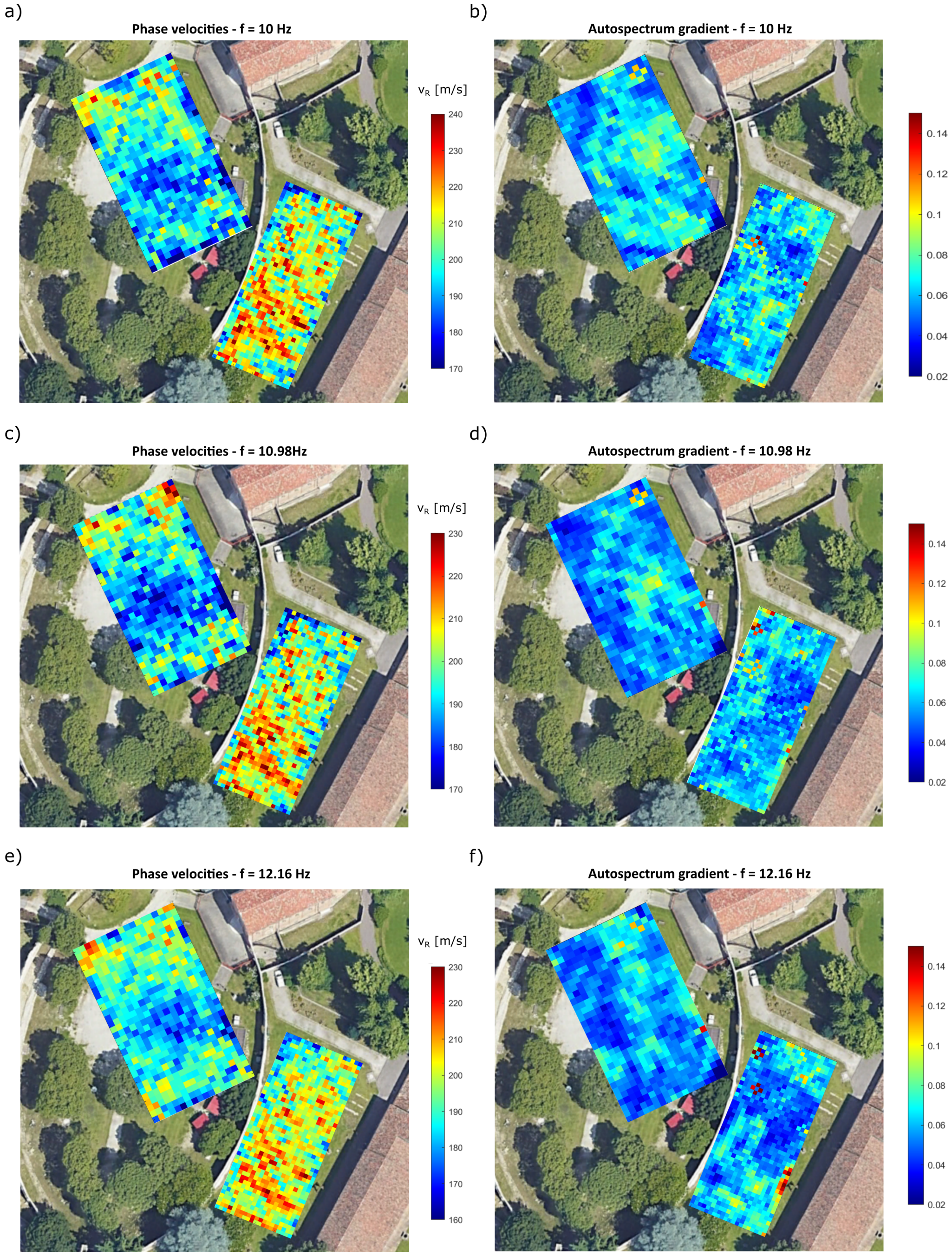


**Figure S6.** Phase velocity maps (a, c, e) and autospectrum gradient maps (b, d, f) for frequencies 10 Hz, 10.98 Hz and 12.16 Hz.

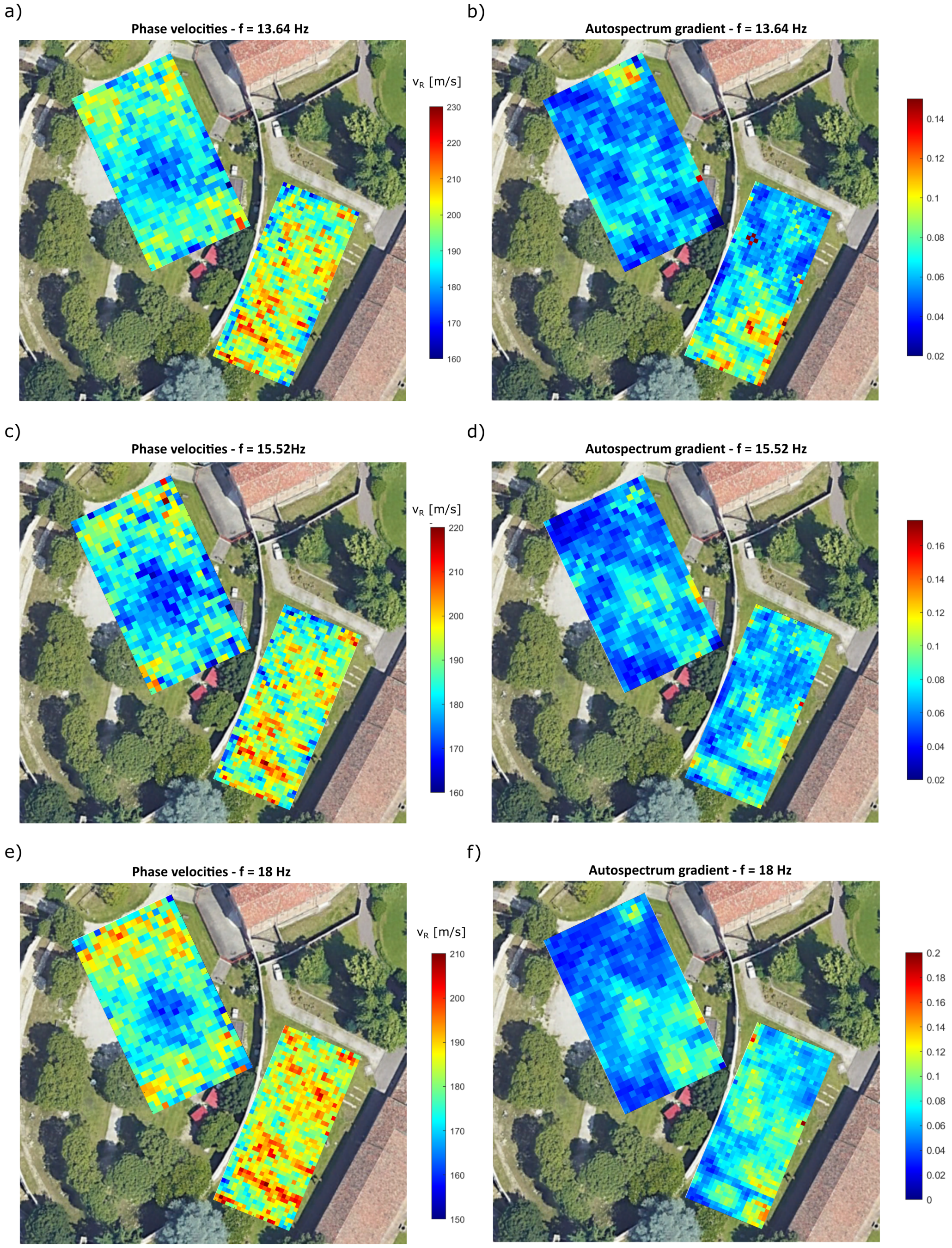


**Figure S7.** Phase velocity maps (a, c, e) and autospectrum gradient maps (b, d, f) for frequencies 13.64 Hz, 15.52 Hz and 18 Hz.

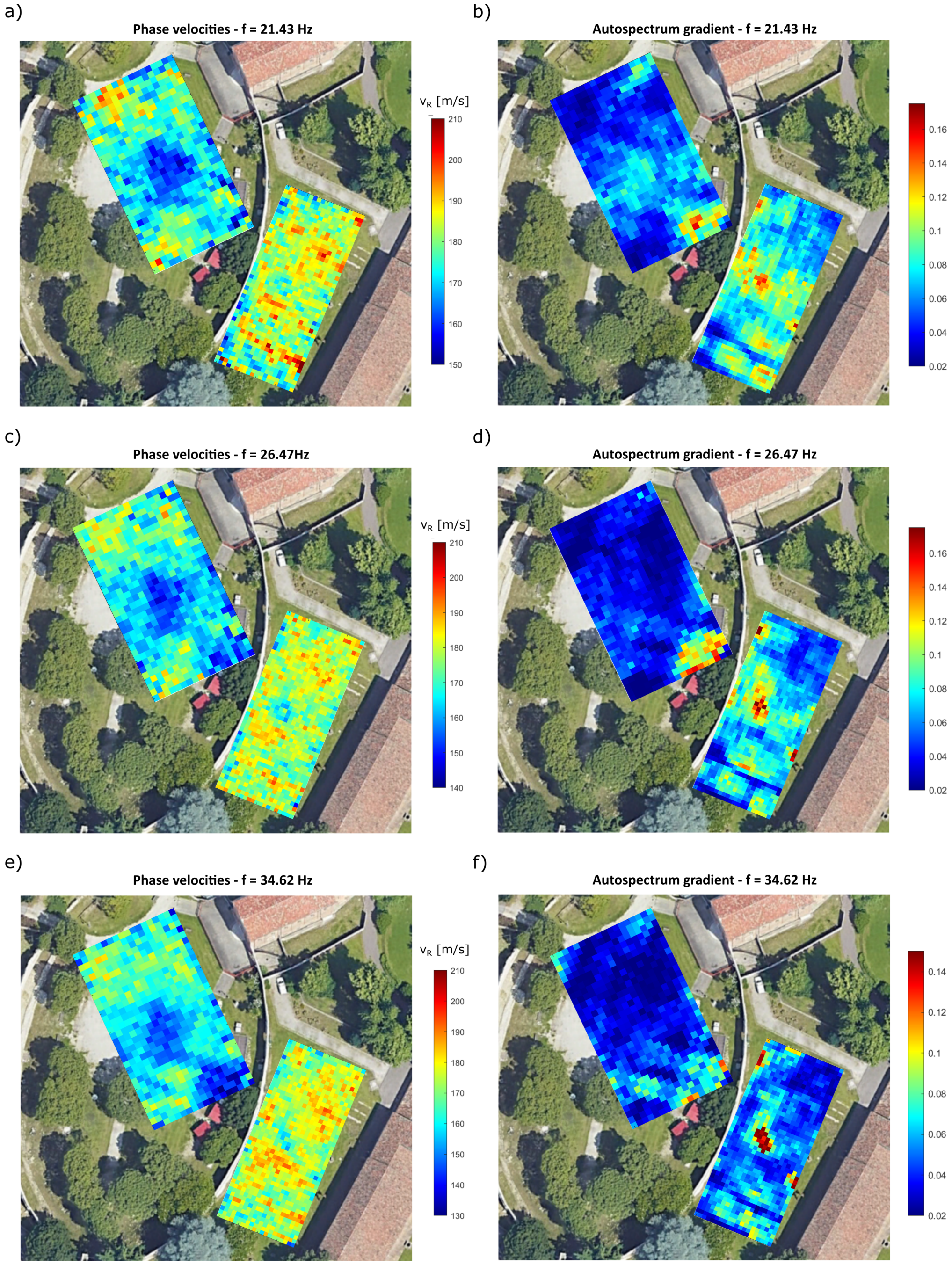


**Figure S8.** Phase velocity maps (a, c, e) and autospectrum gradient maps (b, d, f) for frequencies 21.43 Hz, 26.47 Hz and 34.62 Hz.

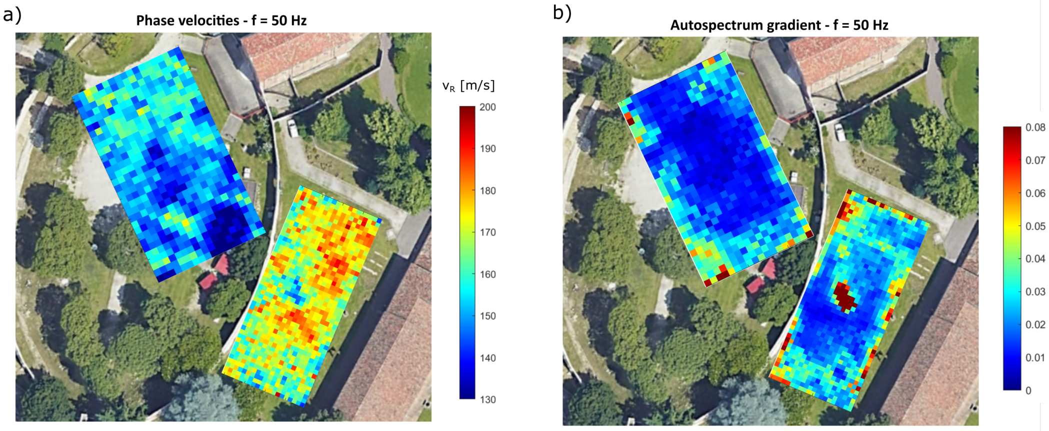


**Figure S9.** Phase velocity map (a) and autospectrum gradient map (b) for the frequency 50 Hz.

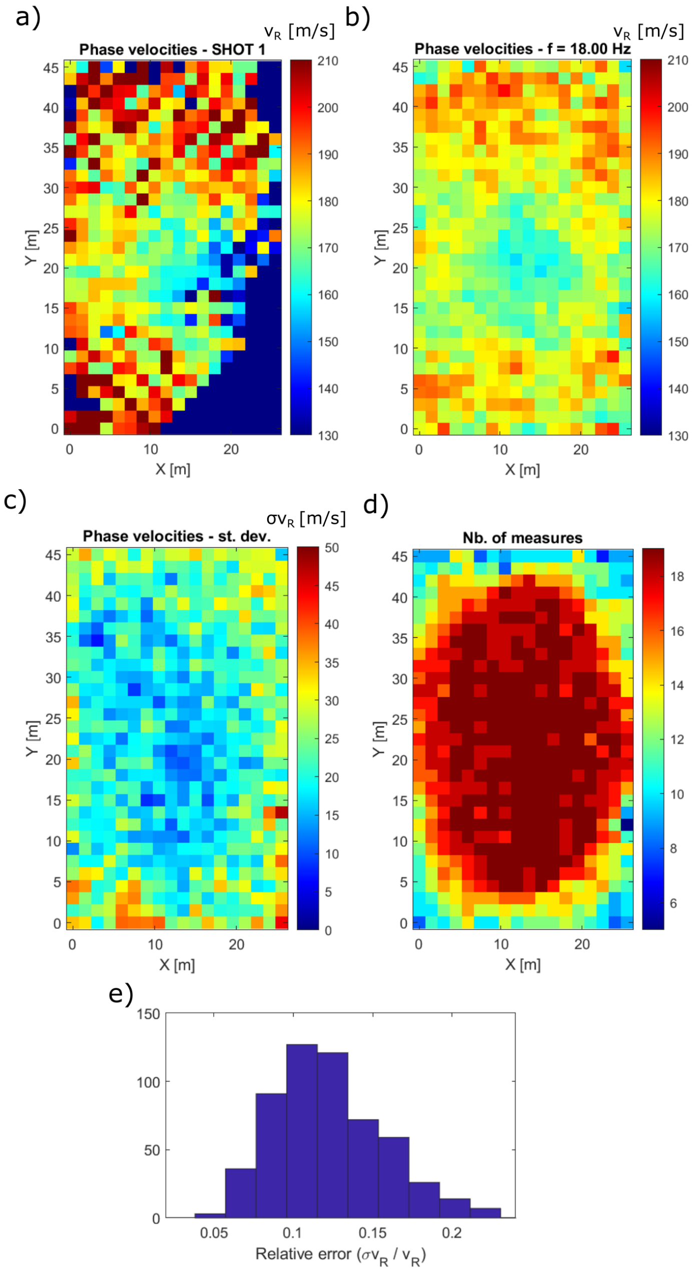


**Figure S10.** Phase velocities and relative uncertainties for 18 Hz and receiver grid 1. X and Y refer to a local coordinate system with its origin at the southernmost receiver. (a) Phase velocity map obtained from shot 1. (b) Final phase velocity map obtained by averaging velocities of shots 1 to 19. (c) Phase velocity standard deviations. (d) Number of measurements used for the average.

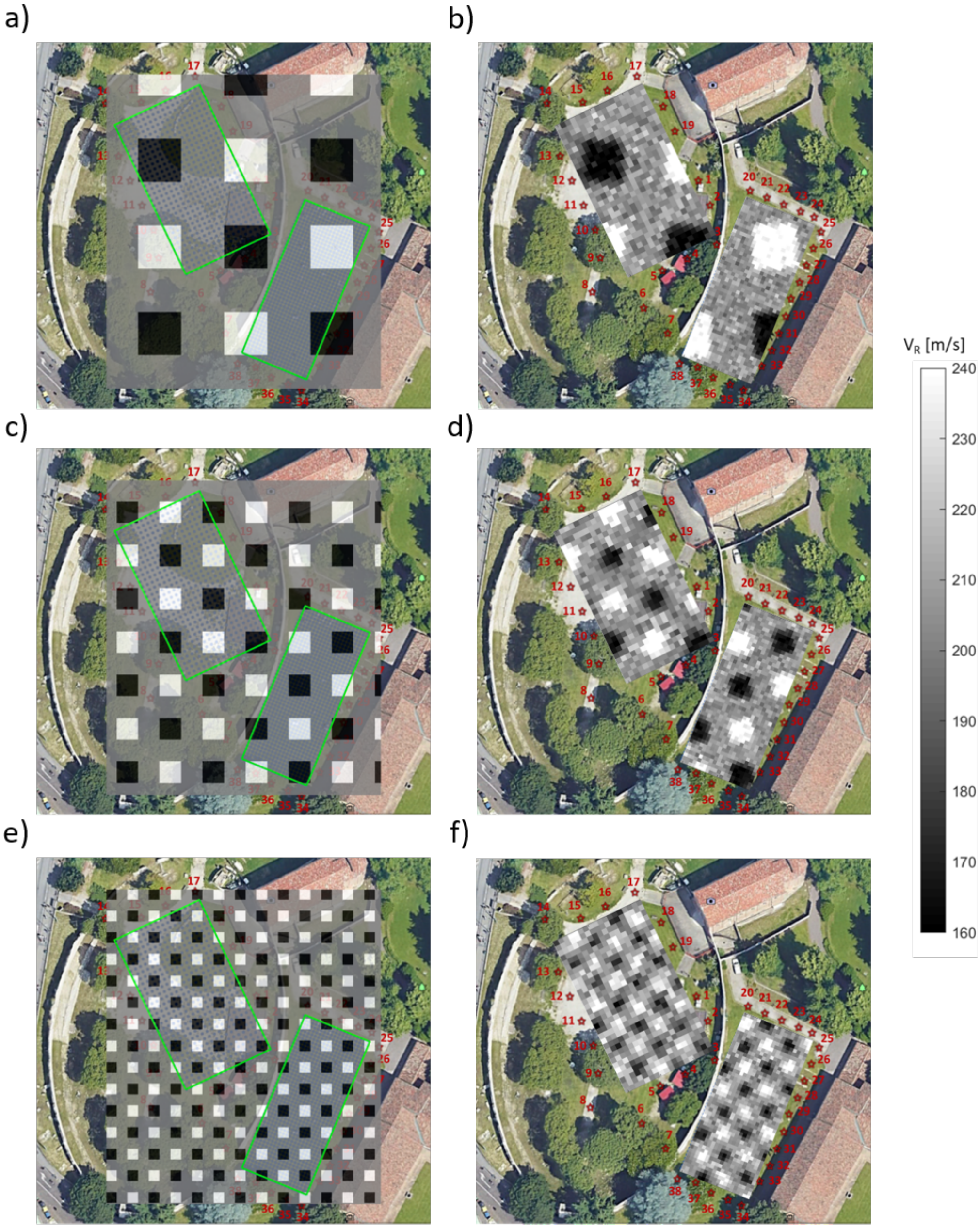


**Figure S11.** Checkerboard tests with velocity anomalies of 20% and realistic noise added to phase slownesses. On the left: input synthetic models. On the right: models output by the Eikonal tomography. The size of the anomalies is 12 m (a, b), 6 m (c, d) and 3 m (e, f).

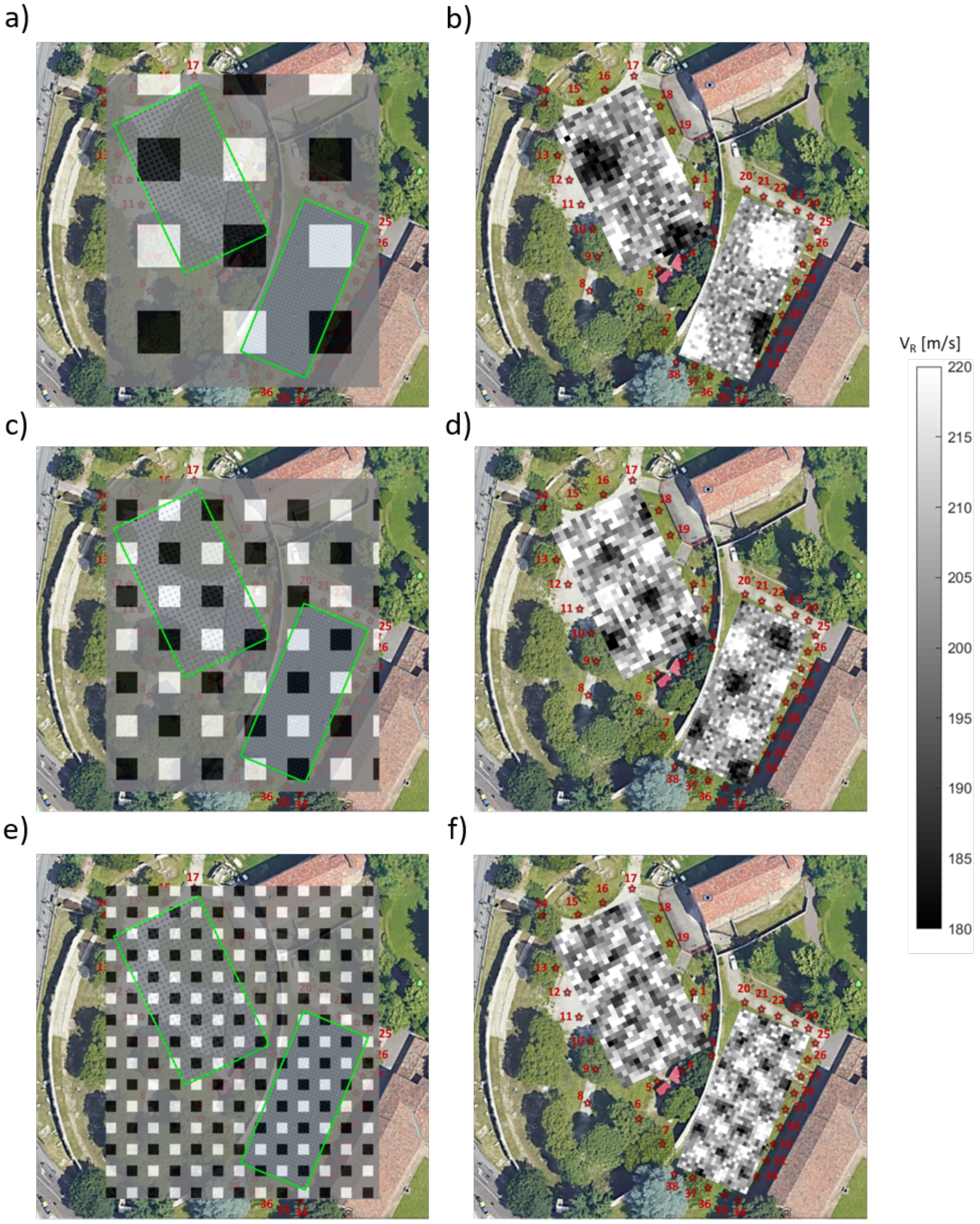


**Figure S12.** Checkerboard tests with velocity anomalies of 10% and realistic noise added to phase slownesses. On the left: input synthetic models. On the right: models output by the Eikonal tomography. The size of the anomalies is 12 m (a, b), 6 m (c, d) and 3 m (e, f).

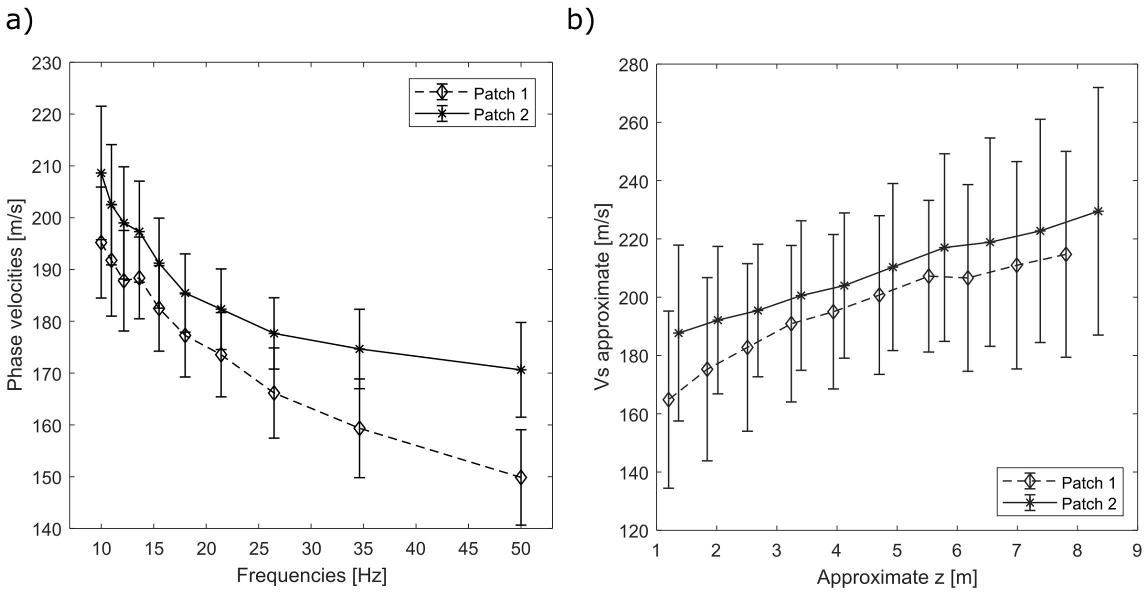


**Figure S13.** (a) Average phase velocities for grid 1 and grid 2 and all frequencies of analysis. (b) Approximated $V_s$, computed as $1.1 \cdot V_r$ ($V_r$ being the average phase velocity), as a function of the approximated depth, obtained as $z = \lambda/2.5 = V_r/(2.5 \cdot f)$ ($\lambda$ being the average wavelength), for grid 1 and grid 2. The curves in (b) were used to define the model parameters in Table S1.

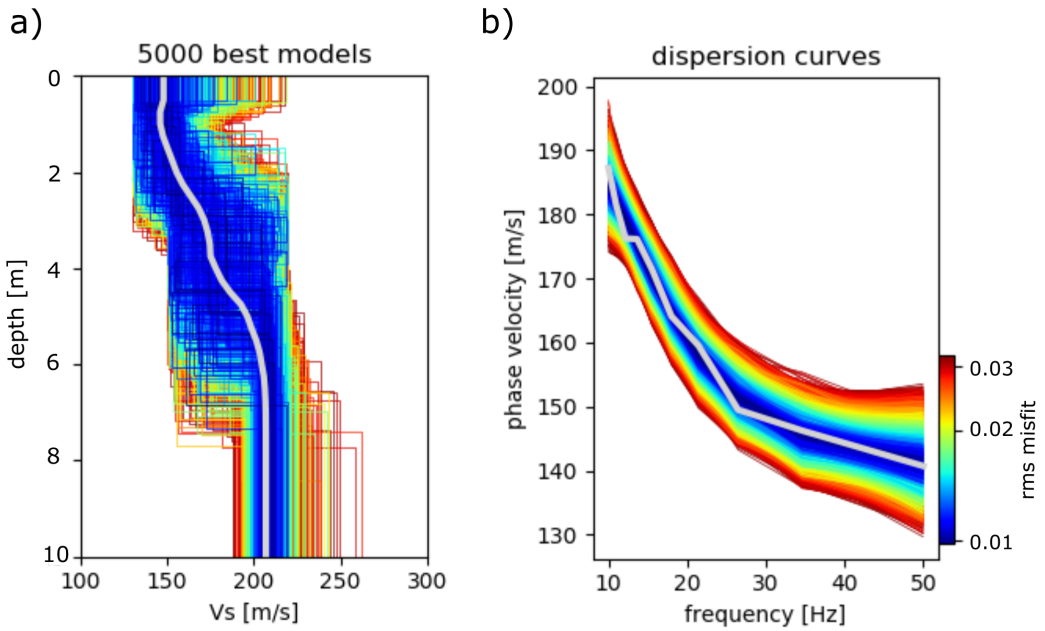


**Figure S14.** Stochastic inversion of one local dispersion curve: (a) 5000 minimum-misfit models (colored lines) and final model (thick grey line), obtained as the average of the best 1000 models. (b) Dispersion curves associated with the models in (a) (in color), and observed curve (in grey).
